# Aquatic Exercise as a Complementary Intervention for Cognitive, Behavioral, Motor, and Functional Outcomes in Attention-Deficit/Hyperactivity Disorder, Autism Spectrum Disorder, and Down Syndrome: A Narrative Review

**DOI:** 10.3390/jcm15145334

**Published:** 2026-07-08

**Authors:** Felipe Montalva-Valenzuela, Eduardo Guzmán-Muñoz, Exal Garcia-Carrillo, Claudio Farias-Valenzuela, Joaquín González-Aroca, Yeny Concha-Cisternas, Iván Molina-Márquez, Rodrigo Yañez-Sepúlveda, Antonio Castillo-Paredes

**Affiliations:** 1 Escuela de Entrenador en Actividad Física y Deporte, Facultad de Ciencias Humanas, Universidad Bernardo O’Higgins, Santiago 8370040, Chile; felipe.montalva@ubo.cl; 2 Escuela de Kinesiología, Facultad de Salud, Universidad Santo Tomás, Talca 3460000, Chile; yenyconchaci@santotomas.cl; 3 Escuela de Fonoaudiología, Facultad de Ciencias de la Salud, Universidad Autónoma de Chile, Talca 3460000, Chile; 4 Department of Physical Activity Sciences, Faculty of Education Sciences, Universidad Católica del Maule, Talca 3480112, Chile; exal.garcia@gmail.com; 5 Department of Physical Activity Sciences, Universidad de Los Lagos, Osorno 5290000, Chile; 6 Escuela de Ciencias de la Actividad Física, el Deporte y la Salud, Universidad de Santiago de Chile (USACH), Santiago 9170022, Chile; claudio.farias.v@usach.cl; 7 Escuela de Kinesiología, Facultad de Salud, Universidad Santo Tomás, La Serena 1720236, Chile; jgonzalez180@santotomas.cl; 8 Vicerrectoría de Investigación e Innovación, Universidad Arturo Prat, Iquique 1100000, Chile; 9 Pedagogía en Educación Física, Facultad de Educación, Universidad Adventista de Chile, Chillán 3780000, Chile; ivanmolina@unach.cl; 10 Programa Doctorado en Ciencias de la Actividad Física, Universidad Católica del Maule, Talca 3460000, Chile; 11 Escuela de Ciencias del Deporte, Facultad de Educación y Humanidades, Universidad Andres Bello, Viña del Mar 2200055, Chile; rodrigo.yanez.s@unab.cl; 12 School of Medicine, Universidad Espíritu Santo, Samborondón 092301, Ecuador; 13 Grupo AFySE, Investigación en Actividad Física y Salud Escolar, Escuela de Pedagogía en Educación Física, Facultad de Educación, Universidad de Las Américas, Santiago 8370040, Chile; acastillop85@gmail.com

**Keywords:** aquatic exercise, swimming, neurodevelopmental disorders, attention-deficit/hyperactivity disorder, autism spectrum disorder, Down syndrome, physical activity

## Abstract

**Background**: Neurodevelopmental disorders such as attention-deficit/hyperactivity disorder (ADHD), autism spectrum disorder (ASD), and Down syndrome (DS) are associated with cognitive, behavioral, motor, and functional impairments that may negatively affect daily functioning and quality of life. Aquatic exercise has emerged as a potential complementary intervention due to its unique physical and sensory characteristics. **Objective**: To analyze the available evidence regarding aquatic exercise as a complementary intervention for individuals with ADHD, ASD, and DS, considering its potential effects on cognitive, behavioral, motor, and functional outcomes. **Methods**: A narrative review was conducted using studies identified in PubMed, Scopus, and Web of Science between November 2025 and February 2026. Experimental and quasi-experimental designs, including case studies, evaluating aquatic exercise interventions in individuals with ADHD, ASD, or DS were included. **Results**: Twenty-two studies were analyzed (ADHD = 7, ASD = 10, DS = 5). In ADHD, aquatic exercise was associated with improvements in inhibitory control, attention, cognitive flexibility, behavioral regulation, academic performance, and cardiorespiratory fitness. In ASD, the main benefits included improvements in balance, motor coordination, aquatic skills, social interaction, communication, adaptive behavior, and behavioral regulation, particularly in Halliwick-based interventions. In DS, positive effects were mainly observed in aerobic capacity, muscular strength, body composition, balance, functional physical fitness, and motor autonomy. Across studies, interventions commonly involved 2–3 weekly sessions lasting 30–90 min over 6–36 weeks. **Conclusions**: Aquatic exercise appears to be a promising complementary intervention for individuals with ADHD, ASD, and DS, with potential benefits across cognitive, behavioral, motor, and functional domains. Although the available evidence is heterogeneous and methodological limitations remain, aquatic exercise may represent a valuable component of interdisciplinary intervention and adapted physical activity programs.

## 1. Introduction

Neurodevelopmental disorders represent a wide range of conditions characterized by cognitive and developmental impairments that may persist throughout life [1]. These disorders include attention-deficit/hyperactivity disorder (ADHD), intellectual disability (ID), in which Down syndrome (DS) is included, and autism spectrum disorder (ASD), among others [2]. It is estimated that approximately 15% of children worldwide present one of these conditions [3,4]. Due to the impairments associated with neurodevelopmental disorders, individuals tend to experience poor academic performance, difficulties in social skills and daily life functioning [5,6,7], in addition to a significant psychosocial burden for affected individuals, their families, and caregivers [8].

ADHD is characterized by three main symptoms: inattention, hyperactivity, and impulsivity [9]. It is estimated worldwide prevalence ranges from 2% to 18%, with a higher frequency observed in males compared to females, at an approximate ratio of 3:1 [10]. This condition significantly affects cognitive and motor functions, potentially impairing personal, social, and even occupational development [9,11]. Currently, pharmacological treatment remains one of the primary therapeutic strategies for ADHD, showing significant improvements supported by strong scientific evidence [12]. However, in recent years, there has been growing interest in complementary interventions that may contribute to symptom management.

ASD is a neurodevelopmental disorder that has shown an increase in prevalence over recent years [13]. It is characterized by impairments in social interaction, communication difficulties, and the presence of repetitive or stereotyped behaviors and interests [14]. These characteristics are commonly manifested through rigid routines, motor stereotypies, and repetitive behaviors, such as arm flapping, hand movements, or body rocking [15]. Since ASD has no specific cure, its management is generally approached from a multidisciplinary perspective, including pharmacotherapy, music therapy, psychoeducational interventions, and physical activity [16].

DS is a genetic condition caused by trisomy 21 and represents the most common genetic alteration associated with intellectual disability worldwide [17]. Children with DS commonly present hypotonia, ligamentous hyperlaxity, delayed muscle activation, and deficits in postural control [18,19]. In addition, they exhibit a high prevalence of congenital heart disease, considered one of the leading causes of mortality and morbidity in this population [20,21]. These characteristics may negatively affect the quality of life of individuals with DS [22].

Due to the cognitive, motor, and behavioral limitations present in these populations, different complementary strategies have emerged aimed at promoting overall development and improving quality of life. In this context, several studies have reported that physical exercise may generate benefits in individuals with ADHD, ASD, and DS [23,24,25]. In particular, aquatic exercise has gained increasing interest, as it can be performed through a playful approach while achieving similar benefits [26]. Moreover, aquatic exercise is recommended both for healthy individuals and for those with various health conditions [27], and preliminary evidence suggests that it may be safely prescribed for individuals with disabilities [28]. Among its main benefits are low joint impact, improvements in physical fitness, and potential positive effects on inflammatory processes and cardiometabolic risk factors, such as obesity and diabetes [29]. Similarly, improvements in physical fitness, lipid profile, and immune system function have been observed in children with disabilities following aquatic exercise interventions [30].

Although ADHD, ASD, and DS differ substantially in their clinical characteristics, aquatic exercise has emerged as a complementary intervention across all three conditions. Synthesizing the evidence across these populations may facilitate comparison, helping to identify both shared and disorder-specific effects while providing a broader perspective on the potential role of aquatic exercise as a complementary intervention for individuals with neurodevelopmental disorders. Such a synthesis may also encourage future research exploring aquatic exercise in other neurodevelopmental disorders.

Despite the growing interest in aquatic exercise among populations with neurodevelopmental disorders, the available evidence remains heterogeneous and scattered, with limited integration of its potential cognitive, behavioral, and motor effects in ADHD, ASD, and DS. Therefore, the present narrative review aimed to analyze the available evidence regarding aquatic exercise as a complementary intervention for individuals with ADHD, ASD, and DS, considering its potential effects on cognitive, behavioral, motor, and functional outcomes.

## 2. Materials and Methods

A bibliographic search was conducted between November 2025 and February 2026 in three major databases: PubMed, Scopus, and Web of Science. The search strategy was developed in three independent stages, aiming to identify the largest possible number of studies related to each analyzed condition: ADHD, ASD, and DS.

Different combinations of keywords related to aquatic exercise were used for each condition. For ADHD, terms such as “ADHD”, “Attention Deficit”, “swimming”, “aquatic exercise”, “water exercise”, “pool exercise”, “symptomatology”, “executive functions”, “motor skills”, “behavior”, “children”, “adolescent”, “pediatric”, “adult”, and “young adult” were employed. For ASD, the terms used included “Autism”, “ASD”, “autism spectrum disorder”, “swimming”, “aquatic exercise”, “water exercise”, “executive function”, “self-regulation”, “disruptive behavior”, “motor skills”, “social interaction”, “children”, “adolescent”, “adult”, and “young adult”. Finally, for Down syndrome, the terms “Down Syndrome”, “Trisomy 21”, “swimming”, “aquatic exercise”, “water exercise”, “cognitive skills”, “motor development”, “physical fitness”, “health”, “children”, “adolescent”, “adult”, and “young adult” were used.

The searches were conducted using Boolean operators (“AND” and “OR”), adapting the search strategies according to the characteristics of each database. No restrictions on publication year were applied, and only articles published in English were considered. The complete search strategies for each database are provided in Supplementary Table S1. In addition, a manual review of the reference lists of the selected studies was performed to identify potentially relevant additional publications for this review.

Studies using aquatic exercise as the main intervention and evaluating potential improvements in variables associated with the clinical, cognitive, behavioral, or motor characteristics of the analyzed populations were considered eligible. Experimental and quasi-experimental studies were included, such as randomized controlled trials, non-randomized studies, pre-experimental and quasi-experimental designs, and case reports, provided they explicitly described an intervention developed in an aquatic environment. Observational studies, reviews, and publications that did not adequately describe the intervention were excluded.

Initially, titles and abstracts were reviewed to determine their potential relevance. Subsequently, the selected studies were analyzed through full-text reading to define their final inclusion in the review.

The collected evidence was analyzed and synthesized considering the possible mechanisms associated with the benefits of aquatic exercise, as well as the characteristics of the reported interventions, including weekly frequency, session duration, total intervention time, and the main outcomes observed in the different populations studied.

## 3. Results

### 3.1. Aquatic Exercise in People with ADHD

**Figure 1:**
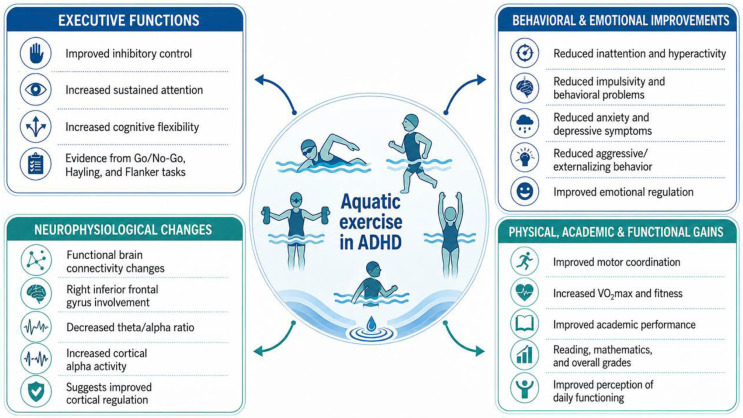
Effects of aquatic exercise in individuals with ADHD. This figure summarizes the main reported effects of aquatic exercise in individuals with attention-deficit/hyperactivity disorder (ADHD). The evidence suggests improvements across executive function domains, including inhibitory control, sustained attention, and cognitive flexibility, together with neurophysiological changes related to functional brain connectivity, theta/alpha ratio, cortical alpha activity, and cortical regulation. Behavioral and emotional benefits include reductions in inattention, hyperactivity, impulsivity, anxiety, depressive symptoms, and aggressive or externalizing behaviors. Physical, academic, and functional gains include improvements in motor coordination, cardiorespiratory fitness, academic performance, and perceived daily functioning.

The available evidence regarding the application of aquatic exercise in individuals with ADHD suggests positive effects across multiple domains of cognitive, behavioral, emotional, and motor functioning [31,32,33,34,35,36,37]. Collectively, the findings converge in showing that the main effects are observed in the domain of executive functions, particularly inhibitory control, followed by improvements in sustained attention and cognitive flexibility (Figure 1). Across the reviewed studies, consistent reductions in symptoms of inattention and hyperactivity were observed, together with decreases in behavioral problems, anxiety, depression, and impulsivity, as well as improvements in the perception of daily and occupational functioning [31,34,37]. Additionally, some studies reported reductions in externalizing problems, aggressive behavior, and social difficulties, along with improvements in emotional regulation and behavioral adaptation following aquatic interventions [34,37].

Regarding executive functions, significant improvements in inhibitory control have been reported, evidenced through Go/No-Go, Hayling, and Flanker tasks, showing reduced response time, greater accuracy, and fewer inhibitory errors after swimming and aerobic aquatic exercise programs [32,34,35]. Ding et al. [32] additionally described changes in functional brain connectivity, particularly in the right inferior frontal gyrus, a region associated with self-regulation and behavioral inhibition processes. Similarly, Chang et al. [35] observed specific improvements in accuracy during NoGo stimuli, suggesting a direct effect of aquatic exercise on response inhibition mechanisms.

From a broader cognitive perspective, studies also report improvements in cognitive flexibility, selective attention, and overall executive performance [33,34]. Silva et al. [33] found significant improvements in cognitive flexibility and selective attention, accompanied by reductions in stress and depression in adolescents with ADHD following a swimming learning program.

Neurophysiological evidence also supports behavioral findings. Huang et al. [36] reported reductions in the theta/alpha ratio in frontal and central regions following a water aerobics program, along with increased cortical alpha activity, suggesting improved cortical regulation associated with attentional and inhibitory control. These findings are consistent with the observed behavioral performance changes and support the hypothesis of a partial normalization of neurophysiological patterns associated with ADHD following aquatic exercise [32,36].

At the functional and physical level, positive outcomes have been observed in academic performance, including reading, mathematics, and overall grade average, as well as significant increases in VO_2_max and enhancements in motor coordination, laterality, hand-eye skills, flexibility, and abdominal endurance [33,34,35]. Hattabi et al. [34] described simultaneous functional gains in inhibitory control, academic performance, and cardiorespiratory fitness following 12 weeks of adapted recreational swimming. Similarly, Chang et al. [35] documented improvements in coordinative motor skills, while Silva et al. [33] reported enhancements in lower-limb coordination and laterality.

Regarding intervention characteristics, the evidence includes diverse aquatic exercise formats, such as intensive swimming programs with complementary dry-land training, moderate aerobic swimming, swimming learning programs with aquatic adaptation, recreational swimming with aquatic games, water aerobics programs with perceptual-motor components, and water treadmill running, all varying in duration, intensity, and intervention focus [31,32,33,34,35,36,37]. This methodological heterogeneity limits direct comparison between studies, although the effects tend to be consistent in the direction of improvement.

Mechanistically, the potential effects of aquatic exercise may be explained by the interaction between the benefits of aerobic exercise and the specific properties of the aquatic environment. Physical exercise has been associated with modulation of dopaminergic and noradrenergic systems, together with increases in neurotrophic factors such as BDNF, favoring neuroplasticity processes linked to executive control and attention [38,39]. In turn, the physical and sensory properties of the aquatic environment may promote behavioral and emotional self-regulation in individuals with ADHD.

In practical terms, these findings position aquatic exercise as a complementary alternative within non-pharmacological ADHD interventions, especially in educational, therapeutic, or community settings where adherence to physical activity is sought through structured, safe, and playful approaches.

### 3.2. Aquatic Exercise in People with ASD

**Figure 2:**
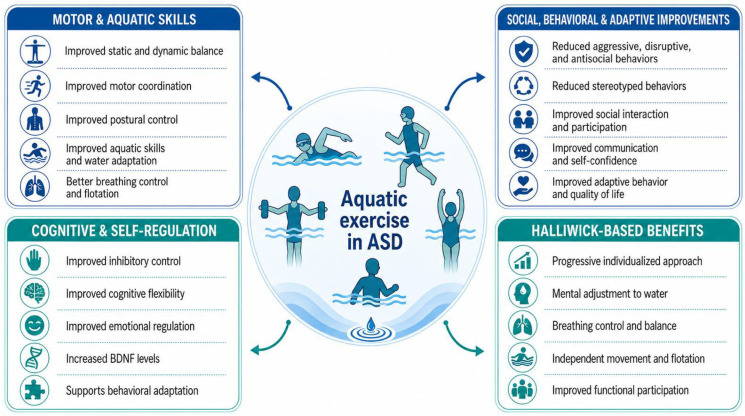
Effects of aquatic exercise in individuals with ASD. This figure summarizes the main reported effects of aquatic exercise in individuals with autism spectrum disorder (ASD). Aquatic interventions, including adapted swimming, hydrotherapy, recreational aquatic exercise, and Halliwick-based programs, have been associated with improvements in motor and aquatic skills, including balance, coordination, postural control, water adaptation, breathing control, and flotation. Reported benefits also include improvements in social interaction, communication, participation, self-confidence, adaptive behavior, and quality of life, as well as reductions in aggressive, disruptive, antisocial, and stereotyped behaviors. Some evidence also suggests favorable effects on inhibitory control, cognitive flexibility, emotional regulation, and BDNF levels.

The reviewed evidence suggests that aquatic exercise constitutes a complementary strategy with favorable effects across multiple dimensions of functioning in children with ASD. The different analyzed programs (including adapted swimming, hydrotherapy, recreational aquatic exercise, and especially interventions based on the Halliwick method) report motor, behavioral, social, and functional benefits, positioning the aquatic environment as a potentially facilitating setting for this population (Figure 2) [40,41,42,43,44,45,46,47,48,49].

Several studies indicate that children with ASD commonly present difficulties in gross motor skills, balance, coordination, postural control, motor planning, and participation in physical activity. In this context, the physical and sensory properties of water may facilitate motor exploration, participation, and motor learning in children with greater functional difficulties or less sports experience.

Regarding the motor domain, studies report consistent improvements in static and dynamic balance, motor coordination, postural control, aquatic skills, and functional capacities. Ansari et al. [48] documented significant gains in static and dynamic balance following a ten-week aquatic program, while Lotan & Weiss [47] reported advances in balance, coordination, and manual dexterity after an individualized hydrotherapy program. Similarly, Vodakova et al. [49] demonstrated improvements in mental adjustment to water, breathing control, flotation, and gross motor function assessed through the GMFM. Likewise, Fragala-Pinkham et al. [42] found within-group improvements in aquatic skills and functional performance, although without significant differences compared to the control group.

The benefits described in the literature are not restricted solely to motor performance. Several studies report improvements in social interaction, communication, participation, and adaptive behavior following aquatic programs. Pan [44] observed reductions in aggressive, disruptive, and antisocial behaviors, together with improvements in social skills, participation, and self-confidence. Similarly, Güeita-Rodríguez et al. [40] reported advances in social acceptance, psychosocial functioning, and quality of life, in addition to parent-perceived improvements in communication and behavior. Marzouki et al. [46] also reported significant reductions in stereotyped behaviors following technical and recreational aquatic interventions.

At the cognitive level, some studies suggest favorable effects on executive functions and emotional regulation. Zhao et al. [43] reported significant improvements in inhibitory control and cognitive flexibility, together with increases in BDNF levels following a 12-week aquatic program, although no significant changes were observed in working memory. These findings suggest that aquatic exercise may influence not only motor performance, but also neurocognitive processes related to self-regulation and behavioral adaptation.

Among aquatic interventions, the Halliwick method stands out due to its progressive and individualized approach. Programs based on this method, such as those developed by Kemp et al. [41], Pan [44], and Vodakova et al. [49], demonstrate relevant improvements in aquatic skills, water adaptation, and functional participation. Its gradual structure, focused on mental adjustment to water, breathing control, balance, and independent movement, appears especially suitable for children with ASD, as it allows activities to be adapted according to the sensory, motor, and behavioral needs of each participant.

Nevertheless, some studies show that certain skills continue to be difficult to develop, especially those related to full immersion, advanced breathing control, or independent flotation [41,49]. Vodakova et al. [49] observed that the greatest progress occurred during the first weeks of intervention, followed by a stabilization phase, while Fragala-Pinkham et al. [42] suggested that low-intensity programs may limit the magnitude of the observed improvements. Similarly, Koumenidou et al. [45] described difficulties related to motivation and concentration during aquatic sessions. Taken together, these findings suggest the need for longer, more structured interventions with continuous follow-up.

Overall, the available evidence suggests that aquatic exercise, particularly through Halliwick-based interventions, may contribute to motor development, behavioral regulation, sensory adaptation, social interaction, and functional abilities in children with ASD, representing a potentially useful complementary alternative within multidisciplinary intervention programs.

### 3.3. Aquatic Exercise in People with DS

**Figure 3:**
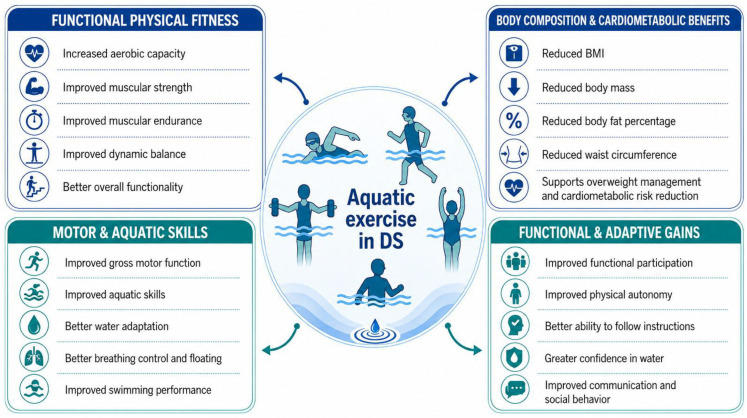
Effects of aquatic exercise in individuals with DS. This figure summarizes the main reported effects of aquatic exercise in individuals with Down syndrome (DS). The available evidence suggests that structured swimming, hydrotherapy, and progressive aquatic training may improve functional physical fitness, including aerobic capacity, muscular strength, muscular endurance, dynamic balance, and overall functionality. Aquatic exercise may also enhance gross motor function, aquatic skills, water adaptation, breathing control, floating, swimming performance, functional participation, physical autonomy, confidence in water, communication, and social behavior. In addition, long-term aquatic programs have been associated with favorable changes in body composition and cardiometabolic indicators, including reductions in BMI, body mass, body fat percentage, and waist circumference.

The available evidence on aquatic exercise in individuals with DS suggests broad positive effects on physical functioning, particularly in the development of functional physical fitness, motor skills, and body composition. The reviewed studies include structured swimming programs, hydrotherapy, and progressive aquatic training conducted in children, adolescents, and adults with DS. The findings show consistent improvements in aerobic capacity, muscular strength, balance, aquatic skills, functionality, and anthropometric variables, suggesting a comprehensive impact on different components of functional physical fitness (Figure 3) [50,51,52,53,54].

In pediatric populations, group-based aquatic programs focused on motor learning and water adaptation have demonstrated relevant benefits in gross motor skills, aquatic competencies, and functional participation. Milligan et al. [50] reported significant improvements in gross motor function and aquatic skills following an eight-week group program based on floating exercises, breathing control, balance, and sensory activities. In addition to the changes observed in GMFM and WOTA-1 assessments, caregivers described improvements in following instructions, confidence in the water, communication, and social behavior, suggesting functional benefits beyond the physical domain.

In adolescents with DS, studies report particularly positive effects on aerobic capacity and body composition. Naczk et al. [51] observed that a 33-week progressive swimming program produced significant increases in VO_2_max, muscular strength, and endurance, together with reductions in BMI, body mass, and body fat percentage. Similarly, Suarez-Villadat et al. [53] reported significant decreases in adiposity indicators, including BMI, waist circumference, and body fat percentage, following a 36-week swimming program performed three times per week. These findings indicate that long-term aquatic interventions may contribute to overweight management and improvements in cardiometabolic risk factors, which are frequently observed in this population.

In adults with DS, aquatic programs also demonstrate relevant improvements in functional fitness and physical autonomy. Boer & de Beer [52] found significant increases in aerobic capacity, muscular strength, and overall functionality after six weeks of aquatic training, although without substantial changes in static balance or body composition. Likewise, Boer [54] demonstrated that an eight-week freestyle swimming training program produced improvements in dynamic balance, muscular strength, functional capacity, swimming performance, and anthropometric variables, with moderate-to-large effect sizes across several functional measures.

In summary, the available evidence suggests that aquatic exercise represents a promising strategy for improving functionality and performance capacity in individuals with DS, although the literature remains limited in the number of specific studies. Its implementation within adapted physical activity programs is mainly supported by its potential to integrate improvements across multiple physical capacities within a single training stimulus.

### 3.4. Comparative Analysis of the Evidence in ADHD, ASD, and DS

In general, the reviewed evidence suggests that aquatic exercise constitutes a complementary strategy with consistent benefits in individuals with ADHD, ASD, and DS, although important differences exist regarding intervention focus and the variables evaluated. In studies involving ADHD and ASD, aquatic interventions are primarily oriented toward cognitive, behavioral, and self-regulation-related outcomes, whereas in DS, the emphasis is mainly placed on the development of functional physical fitness and motor capacity. In ADHD, programs report improvements in inhibitory control, attention, cognitive flexibility, behavioral regulation, emotional symptoms, and academic performance, in some cases accompanied by neurophysiological and functional connectivity changes associated with executive functions [31,32,33,34,35,36,37]. Similarly, in ASD, the predominant benefits are related to balance, motor coordination, aquatic skills, behavioral regulation, social interaction, communication, and functional participation, particularly in interventions based on the Halliwick method and adapted playful approaches [40,41,42,43,44,45,46,47,48,49]. In contrast, studies involving individuals with DS show more consistent improvements in VO_2_max, muscular strength, dynamic balance, body composition, muscular endurance, and overall functionality, reflecting a stronger orientation toward functional fitness and physical autonomy [50,51,52,53,54].

**Figure 4:**
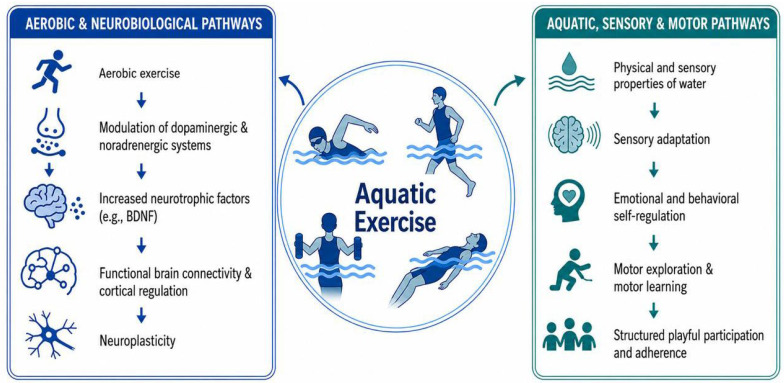
Conceptual framework illustrating the potential mechanisms through which aquatic exercise may influence cognitive, behavioral, motor, and functional outcomes in individuals with ADHD, ASD, and DS. This figure presents a conceptual synthesis of the proposed mechanisms through which aquatic exercise may influence functioning across ADHD, ASD, and DS. Two complementary pathways are proposed. First, the aerobic and neurobiological pathway suggests that aquatic exercise may modulate dopaminergic and noradrenergic systems, increase neurotrophic factors such as BDNF, support functional brain connectivity and cortical regulation, and promote neuroplasticity. Second, the aquatic, sensory, and motor pathway highlights the role of water’s physical and sensory properties in facilitating sensory adaptation, emotional and behavioral self-regulation, motor exploration, motor learning, structured participation, and adherence. These pathways represent a conceptual framework derived from the available evidence and should not be interpreted as experimentally validated mechanisms.

Despite these differences, several common elements can be identified across the three populations. Most interventions were conducted two to three times per week, with sessions ranging approximately from 30 to 90 min and total intervention durations between 6 and 36 weeks, although programs lasting between 8 and 12 weeks were the most frequent. Improvements in motor and functional variables were also observed across all groups, suggesting that the aquatic environment may support not only physical development but also participation, adherence, and self-regulation processes. Another transversal aspect was the high acceptance of the interventions and the good adherence reported by participants and families, especially in programs including recreational activities, aquatic games, and individualized adaptation strategies [40,41,44,50]. In several studies, parents and caregivers described perceived improvements in behavior, participation, confidence, and willingness to engage in physical activity, reinforcing the potential of the aquatic environment as a safe, motivating, and sensorily regulated space for individuals with neurodevelopmental disorders [40,44,50]. Taken together, the evidence suggests that the effects of aquatic exercise across ADHD, ASD, and DS may be explained by the interaction between aerobic-neurobiological mechanisms and the sensory-motor properties of the aquatic environment (Figure 4).

## 4. Discussion

This narrative review aimed to analyze the available evidence on aquatic exercise as a complementary intervention in individuals with ADHD, ASD, and DS, considering its effects on cognitive, behavioral, motor, and functional outcomes. Although the available evidence is encouraging, it should be interpreted considering the methodological diversity of the included studies, which comprise randomized controlled trials, quasi-experimental studies, pilot interventions, and case reports. In summary, the studies reviewed indicate that aquatic-based interventions are associated with benefits across cognitive, behavioral, motor, and functional domains, although with differences depending on the clinical and functional characteristics of each population. While in ADHD and ASD the findings are mainly associated with executive functions, behavioral regulation, and social interaction, in DS, the benefits are primarily concentrated in functional physical fitness and motor autonomy [31,32,33,34,35,36,37,38,39,40,41,42,43,44,45,46,47,48,49,50,51,52,53,54].

Regarding ADHD, the observed findings are consistent with previous reviews describing positive effects of physical exercise on executive functions, particularly inhibitory control, selective attention, and cognitive flexibility [23,55]. The analyzed studies support the notion that aquatic interventions may produce benefits comparable to those reported in land-based programs, while additionally incorporating specific features of the aquatic environment, such as reduced gravitational demands, high motor engagement, and a playful context that may enhance adherence and engagement in physical activity. In this context, the observed improvements in inhibitory control, behavioral symptoms, and academic performance may be related both to the neurophysiological effects of aerobic exercise and to the coordinative and attentional demands inherent to aquatic activities [31,32,33,34,35,36,37].

However, the available evidence also shows that some executive functions remain underexplored in this type of intervention. Working memory, considered a central component of executive functioning and frequently impaired in individuals with ADHD [56] has been scarcely assessed in the reviewed studies. This is consistent with previous reviews reporting limited research attention to this cognitive domain within exercise-based interventions for ADHD [55], and it limits a comprehensive understanding of the potential cognitive effects of aquatic exercise, given that most studies have primarily focused on inhibitory control and attention. In this regard, future research should incorporate broader assessments of executive functions, including working memory, considering its relevance for academic performance, cognitive development, and learning processes [57,58], to more fully understand the neurocognitive impact of aquatic interventions.

In ASD, the results are consistent with the literature describing multicomponent benefits of physical exercise on motor skills, adaptive behavior, and social interaction [59,60]. However, the studies included in this review suggest that the aquatic environment may offer specific advantages for this population due to its sensory properties and the structured nature of many interventions. Programs based on the Halliwick method and adapted recreational approaches reported improvements not only in aquatic skills and balance, but also in social participation, self-confidence, and reductions in disruptive or stereotyped behaviors [40,41,44,46,49]. This may be explained by the fact that water provides an environment with reduced gravitational load, greater postural support, and distinct sensory stimulation, facilitating motor exploration and participation in children with sensory processing and behavioral regulation difficulties.

In DS, the observed findings support the usefulness of aquatic exercise for improving functional physical fitness, aerobic capacity, and motor autonomy, consistent with previous reviews on physical exercise in this population [24,61]. Nevertheless, a relevant finding from the included studies was the relatively consistent presence of improvements in body composition and adiposity, observed in three of the four experimental studies analyzed [51,53,54]. This is particularly interesting considering that previous literature on exercise in DS has reported more heterogeneous results regarding anthropometric outcomes and weight control [24,61]. A possible explanation may be related to the relatively longer duration of some aquatic interventions, together with the predominantly aerobic nature of swimming-based programs.

Another interesting aspect observed in the reviewed studies concerns the intensity of aquatic interventions and differences in their focus depending on the studied population. In individuals with ADHD, several studies reported physiological parameters or descriptors of moderate to moderate-vigorous intensity, generally within ranges close to 50–70% of maximal heart rate [32,34,36,37]. Ding et al. [32] used intensities between 60 and 69% of HRmax, Huang et al. [36] between 50 and 60%, and Sabzi et al. [37] between 55 and 65%, while Hattabi et al. [34] implemented interventions between 50 and 70% of HRmax. In addition, Skalidou et al. [31] used a subjective perceived exertion of 7–8 on a 0–10 scale, also suggesting a relatively high training stimulus. Taken together, these findings show a tendency toward programs with moderate cardiovascular and aerobic emphasis, likely driven by the interest in inducing neurophysiological adaptations associated with executive functions, inhibitory control, and behavioral regulation. This is consistent with findings on brain connectivity, cortical activation, and cognitive performance reported in several ADHD studies [32,36].

In contrast, in the ASD population most studies did not report objective physiological intensity parameters, showing instead a predominant focus on participation, sensory adaptation, behavioral regulation, and motor learning. Many interventions were delivered through recreational activities, hydrotherapy, and Halliwick-based programs, prioritizing progressive adaptation to the aquatic environment, postural control, social interaction, and functional participation rather than cardiovascular stimulus per se [40,41,44,47,49]. Only Fragala-Pinkham et al. [42] reported intensities close to 50–70% of HRmax, while other authors even noted that participant engagement and motivation influenced session development [45]. This methodological difference may reflect that therapeutic goals in ASD are primarily oriented toward sensory regulation, adaptive behavior, and social interaction rather than physical or cardiovascular performance.

In DS, limited objective monitoring of exercise intensity was also observed. Several studies explicitly acknowledged this as a methodological limitation [51,52,54]. However, swimming and aquatic training programs appeared to adopt a more fitness-oriented approach focused on improving functional capacity. Suarez-Villadat et al. [53] was one of the few studies reporting specific heart rate values during sessions, with approximate ranges between 140 and 180 beats per minute depending on the exercise type and swimming style performed. These findings indicate that although physiological intensity remains poorly reported in the literature on aquatic exercise in neurodevelopmental disorders, there may be relevant differences in prescription goals across populations. While ADHD interventions appear more oriented towards aerobic stimuli linked to neurocognitive benefits, ASD programs are predominantly therapeutic and sensory-focused, and DS interventions tend to prioritize physical conditioning and general functional capacity.

Another relevant aspect relates to common characteristics of the analyzed interventions. Most programs used frequencies of two to three sessions per week, with durations ranging from 30 to 90 min and intervention periods between 6 and 36 weeks. Despite methodological heterogeneity, most studies reported good adherence and high acceptance from participants and families, particularly in programs with recreational, group-based, and progressive components [40,41,42,44,50,52]. These findings suggest that the aquatic environment may promote not only physical, cognitive, and functional benefits but also motivation and sustained engagement in physical activity, which is particularly relevant in populations with difficulties participating in conventional exercise programs.

### 4.1. Limitations and Strengths

The main strengths of this narrative review lie in the breadth of included studies and the diversity of populations and intervention formats analyzed. In contrast to reviews focused exclusively on randomized controlled trials, this work incorporated different experimental and quasi-experimental designs, including case studies, allowing for a broader understanding of the current state of evidence on aquatic exercise in individuals with ADHD, ASD, and DS. Likewise, the specific focus on aquatic interventions enabled a more precise analysis of the potential benefits of the aquatic environment as a complementary strategy for intervention and the promotion of physical activity. Similarly, the inclusion of different stages of life (children, adolescents, and adults) enabled observation of these interventions across age groups and functional levels.

At the same time, this review also presents important limitations that should be considered when interpreting the findings. First, as a narrative review rather than a systematic review, the processes of search, selection, and evidence synthesis involve a greater interpretative component and a lower level of methodological control. No meta-analysis was conducted, preventing precise quantification of the magnitude of effects across the analyzed variables. Another relevant limitation is the high heterogeneity of the included interventions, in terms of duration, frequency, intensity, and type of aquatic exercise, which makes it difficult to establish an optimal intervention context to maximize benefits for each population. Furthermore, studies were synthesized according to the objectives of this narrative review rather than being stratified by study design or level of evidence. Consequently, findings from randomized controlled trials, quasi-experimental studies, pilot interventions, and case reports were discussed collectively to provide a comprehensive overview of the available literature. Therefore, the overall conclusions should be interpreted considering the diversity of study designs included in this review. Additionally, there was a tendency toward relatively small sample sizes in many of the included studies, particularly in pilot investigations, quasi-experimental designs, and case studies, which limits the generalizability of the findings and suggests cautious interpretation of the results.

Finally, most studies did not report sex-specific analyses, limiting the understanding of potential differences in response to aquatic exercise between males and females. Similarly, key clinical variables were not consistently considered, such as ADHD subtypes, levels of severity within ASD, or cognitive and functional levels in individuals with DS, all of which may significantly influence responses to the interventions. Lastly, due to the narrative nature of this review, no formal methodological quality assessment or risk of bias evaluation was applied to the included studies; therefore, the findings should be interpreted primarily as a descriptive synthesis of the current state of evidence on aquatic exercise.

### 4.2. Future Lines of Investigation

Future research on aquatic exercise in individuals with ADHD, ASD and DS should focus on more robust methodological designs, incorporating larger sample sizes, longitudinal follow-ups, and analyses stratified by sex and specific clinical characteristics of each population, such as ADHD subtypes, levels of support or diagnostic severity in ASD, and functional and intellectual level in DS.

Furthermore, there is a need to standardize and more precisely monitor exercise intensity variables, particularly in ASD and DS populations, to identify potential “optimal ranges” of intensity associated with greater benefits. In ADHD, future studies should expand the assessment of executive functions, particularly working memory, as well as explore higher-intensity aquatic interventions to determine whether they are associated with additional cognitive benefits.

In addition, it would be relevant to include assessments related to parental and caregiver perceptions, self-perceived physical fitness, and baseline levels of physical activity, considering that these factors may influence the magnitude of improvements observed following aquatic interventions.

### 4.3. Practical Recommendations

Based on the currently available evidence, aquatic exercise may be considered a useful complementary strategy within therapeutic, educational, and adapted physical activity programs for individuals with ADHD, ASD, and DS. The following practical recommendations should be interpreted as preliminary guidance, given the heterogeneity of the interventions included, study designs, and populations. Overall, the studies suggest that interventions performed two to three times per week, with sessions lasting 45 to 90 min and a minimum duration of approximately eight weeks, may generate benefits across motor, behavioral, cognitive, and functional domains.

In ADHD, interventions may prioritize aerobic and coordinative activities with playful components and attentional demands, given their potential relationship with improvements in executive functions and behavioral regulation. In ASD, programs should be delivered through structured and progressive approaches, incorporating visual support, sensory adaptation, and individualized strategies that facilitate participation and social interaction. In DS, interventions may primarily focus on the development of functional physical fitness, aerobic capacity, muscular strength, and motor autonomy.

Across all populations, the findings suggest that the incorporation of aquatic games, recreational activities, and individualized adaptation strategies may enhance adherence and acceptance of the interventions among both participants and their families. Likewise, considering the specific characteristics and needs of these populations, it is recommended that interventions be supervised by professionals trained in adapted physical activity and interdisciplinary practice.

## 5. Conclusions

The reviewed evidence suggests that aquatic exercise may constitute a beneficial complementary intervention for individuals with ADHD, ASD, and DS, with positive effects observed across cognitive, behavioral, motor, and functional domains. In ADHD, the main benefits were related to executive functions, inhibitory control, and behavioral regulation; in ASD, to motor skills, social interaction, and adaptive behavior; whereas in DS, improvements were primarily observed in functional physical fitness and motor autonomy.

Overall, the findings suggest that the physical and sensory properties of the aquatic environment, combined with the effects of aerobic exercise, may facilitate participation, adherence, and functional improvement across different neurodevelopmental conditions. However, due to the methodological heterogeneity of the included studies and the inherent limitations of narrative review design, the results should be interpreted with caution. Nevertheless, the current evidence positions aquatic exercise as a promising complementary intervention capable of promoting multiple cognitive, behavioral, motor, and functional outcomes within interdisciplinary strategies for intervention and adapted physical activity promotion.

## Data Availability

No new data were created or analyzed in this study.

## Supplementary Materials

The following supporting information can be downloaded at: https://www.mdpi.com/article/10.3390/jcm15145334/s1, Table S1: Complete search strategies used in each database.

## Abbreviations

The following abbreviations are used in this manuscript:

ADHD	Attention-Deficit/Hyperactivity Disorder
ASD	Autism Spectrum Disorder
DS	Down Syndrome
BMI	Body Mass Index
BDNF	Brain-Derived Neurotrophic Factor
GMFM	Gross Motor Function Measure
HRmax	Maximal Heart Rate
ID	Intellectual Disability
VO_2_max	Maximal Oxygen Consumption
WOTA	Water Orientation Test Alyn
EEG	Electroencephalography
