# Supplementary material for: Aquatic Exercise as a Complementary Intervention for Cognitive, Behavioral, Motor, and Functional Outcomes in Attention-Deficit/Hyperactivity Disorder, Autism Spectrum Disorder, and Down Syndrome: A Narrative Review
Source: J Clin Med. 2026 Jul 8;15(14):5334. doi: 10.3390/jcm15145334 (PMC13411913; doi:10.3390/jcm15145334)
Supplement: Supplementary file 1 [file jcm-15-05334-s001.zip › jcm-4387970-supplementary.pdf]

Supplementary Table S1. Complete search strategies used in each database.

Database	Population	Search strategy
**PubMed**	ADHD	("Attention Deficit Hyperactivity Disorder"[Mesh] OR ADHD OR "attention-deficit/hyperactivity disorder" OR "Attention Deficit") AND (swimming OR "aquatic exercise" OR "water exercise" OR "pool exercise") AND (symptomatology OR "executive function*" OR "motor skill*" OR behavior*) AND (child* OR adolescen* OR pediatric* OR adult* OR "young adult")
	ASD	("Autism Spectrum Disorder"[Mesh] OR autism OR ASD OR "autism spectrum disorder") AND (swimming OR "aquatic exercise" OR "water exercise" OR "pool exercise") AND ("executive function*" OR "self-regulation" OR "disruptive behavior" OR "motor skill*" OR "social interaction") AND (child* OR adolescen* OR pediatric* OR adult* OR "young adult")
	Down syndrome	("Down Syndrome"[Mesh] OR "Down syndrome" OR "Trisomy 21") AND (swimming OR "aquatic exercise" OR "water exercise" OR "pool exercise") AND ("cognitive skill*" OR "motor development" OR "physical fitness" OR health) AND (child* OR adolescen* OR pediatric* OR adult* OR "young adult")
**Scopus**	ADHD	TITLE-ABS-KEY (("attention deficit hyperactivity disorder" OR ADHD OR "Attention Deficit") AND (swimming OR "aquatic exercise" OR "water exercise" OR "pool exercise") AND (symptomatology OR "executive function*" OR "motor skill*" OR behavior*) AND (child* OR adolescen* OR pediatric* OR adult* OR "young adult"))
	ASD	TITLE-ABS-KEY (("autism spectrum disorder" OR autism OR ASD) AND (swimming OR "aquatic exercise" OR "water exercise" OR "pool exercise") AND ("executive function*" OR "self-regulation" OR "disruptive behavior" OR "motor skill*" OR "social interaction") AND (child* OR adolescen* OR pediatric* OR adult* OR "young adult"))
	Down syndrome	TITLE-ABS-KEY (("Down syndrome" OR "Trisomy 21") AND (swimming OR "aquatic exercise" OR "water exercise" OR "pool exercise") AND ("cognitive skill*" OR "motor development" OR "physical fitness" OR health) AND (child* OR adolescen* OR pediatric* OR adult* OR "young adult"))
**Web of Science**	ADHD	TS=(("attention deficit hyperactivity disorder" OR ADHD OR "Attention Deficit") AND (swimming OR "aquatic exercise" OR "water exercise" OR "pool exercise") AND (symptomatology OR "executive function*" OR "motor skill*" OR behavior*) AND (child* OR adolescen* OR pediatric* OR adult* OR "young adult"))
	ASD	TS=(("autism spectrum disorder" OR autism OR ASD) AND (swimming OR "aquatic exercise" OR "water exercise" OR "pool exercise") AND ("executive function*" OR "self-regulation" OR "disruptive behavior" OR "motor skill*" OR "social interaction") AND (child* OR adolescen* OR pediatric* OR adult* OR "young adult"))
	Down syndrome	TS=(("Down syndrome" OR "Trisomy 21") AND (swimming OR "aquatic exercise" OR "water exercise" OR "pool exercise") AND ("cognitive skill*" OR "motor development" OR "physical fitness" OR health) AND (child* OR adolescen* OR pediatric* OR adult* OR "young adult"))

Search period: November 2025 to February 2026

Databases: PubMed, Scopus, and Web of Science

Language: English only. **Publication period:** No restrictions on publication year were applied.
